# Multifunctional roles of the mammalian CCR4–NOT complex in physiological phenomena

**DOI:** 10.3389/fgene.2014.00286

**Published:** 2014-08-21

**Authors:** Yo-Taro Shirai, Toru Suzuki, Masahiro Morita, Akinori Takahashi, Tadashi Yamamoto

**Affiliations:** ^1^Cell Signal Unit, Okinawa Institute of Science and Technology Graduate UniversityOnna-son, Japan; ^2^Department of Biochemistry, McGill UniversityMontreal, QC, Canada; ^3^Goodman Cancer Research Centre, McGill UniversityMontreal, QC, Canada

**Keywords:** CCR4-NOT, mRNA decay, deadenylation, posttranscriptional regulation, knockout mice

## Abstract

The carbon catabolite repression 4 (CCR4)–negative on TATA-less (NOT) complex serves as one of the major deadenylases of eukaryotes. Although it was originally identified and characterized in yeast, recent studies have revealed that the CCR4–NOT complex also exerts important functions in mammals, -including humans. However, there are some differences in the composition and functions of the CCR4–NOT complex between mammals and yeast. It is noteworthy that each subunit of the CCR4–NOT complex has unique, multifunctional roles and is responsible for various physiological phenomena. This heterogeneity and versatility of the CCR4–NOT complex makes an overall understanding of this complex difficult. Here, we describe the functions of each subunit of the mammalian CCR4–NOT complex and discuss the molecular mechanisms by which it regulates homeostasis in mammals. Furthermore, a possible link between the disruption of the CCR4–NOT complex and various diseases will be discussed. Finally, we propose that the analysis of mice with each CCR4–NOT subunit knocked out is an effective strategy for clarifying its complicated functions and networks in mammals.

## INTRODUCTION

Carbon catabolite repression 4 (CCR4)–negative on TATA-less (NOT) is a versatile complex that regulates various physiological processes in eukaryotes (Collart, 2003). Of its diverse functions, including transcriptional regulation and protein modification, deadenylation of mRNA has been particularly highlighted. Shortening of poly(A) tails leads to the degradation of mRNA via one of two ways, i.e., 5′–3′ digestion by XRN1 after decapping by DCP1–DCP2 or 3′–5′ digestion by the exosome, consequently suppressing gene expression (see Bartlam and Yamamoto, 2010; Doidge et al., 2012a for more details). In mammals, deadenylation is tightly regulated by various enzymes, such as poly(A)-specific ribonuclease (PARN), PAN2–PAN3, Nocturnin, and Angel1/2, as well as CCR4–NOT (Wiederhold and Passmore, 2010; Doidge et al., 2012a). Among these enzymes, PAN2–PAN3 and CCR4–NOT are considered to be dominant in mammalian cells because cytoplasmic mRNA decay is reported to be conducted in two phases: an initial step by PAN2–PAN3 and a subsequent step by CCR4–NOT (Yamashita et al., 2005; Bartlam and Yamamoto, 2010).

*CCR4* was originally identified as a gene required for the regulation of glucose-repressible alcohol dehydrogenase 2 (ADH2) in the yeast *Saccharomyces cerevisiae* (Denis, 1984). The Ccr4p-associated factor (*CAF1*, also known as *POP2*) gene was found to be required for glucose derepression (Sakai et al., 1992). These two gene products were shown to form a large complex with Not proteins, which are related to the regulation of *HIS3* transcription mainly through its TATA-less core promoter (Collart and Struhl, 1993, 1994). The core CCR4–NOT complex consists of Ccr4p, Caf1p, five Not proteins (Not1p–Not5p), Caf40p, and Caf130p in yeast (Chen et al., 2001). Further study revealed that Ccr4p and Caf1p are the major cytoplasmic mRNA deadenylases in yeast (Tucker et al., 2001; see below).

Initially, only the function of the CCR4–NOT complex in transcription was investigated because the core components of the complex were identified as possible transcriptional regulators (Collart, 2003). However, as mentioned above, mRNA silencing by the CCR4–NOT complex was then discovered. Thus, how the CCR4–NOT complex determines target mRNAs for degradation is now one of the more compelling questions. The CCR4–NOT complex interacts with various proteins, including the BTG/Tob family, RNA-binding proteins and miRNA-induced silencing complexes (miRISCs), to specify target mRNAs (Doidge et al., 2012a). Cytoplasmic polyadenylation element-binding protein (CPEB) binds specific mRNAs, to which Tob, as an adapter protein, mediates the recruitment of the CCR4–NOT deadenylase (Hosoda et al., 2011; Ogami et al., 2014). Other RNA-binding proteins, such as Nanos, Pumilio and *fem-3* binding factor (PUF) proteins, Smaug, tristetrapolin (TTP), and Bicaudal-C (Bic-C), were reported to interact with the components of CCR4–NOT and cause the suppression of target mRNAs (Semotok et al., 2005; Goldstrohm et al., 2006, 2007; Chicoine et al., 2007; Suzuki et al., 2010, 2012; Sandler et al., 2011; Van Etten et al., 2012; Fabian et al., 2013; Joly et al., 2013; Bhandari et al., 2014). In addition, CCR4–NOT is involved in the microRNA (miRNA)-mediated deadenylation of mRNAs through interaction with the GW182 proteins (Behm-Ansmant et al., 2006; Braun et al., 2011; Chekulaeva et al., 2011; Fabian et al., 2011).

**FIGURE 1:**
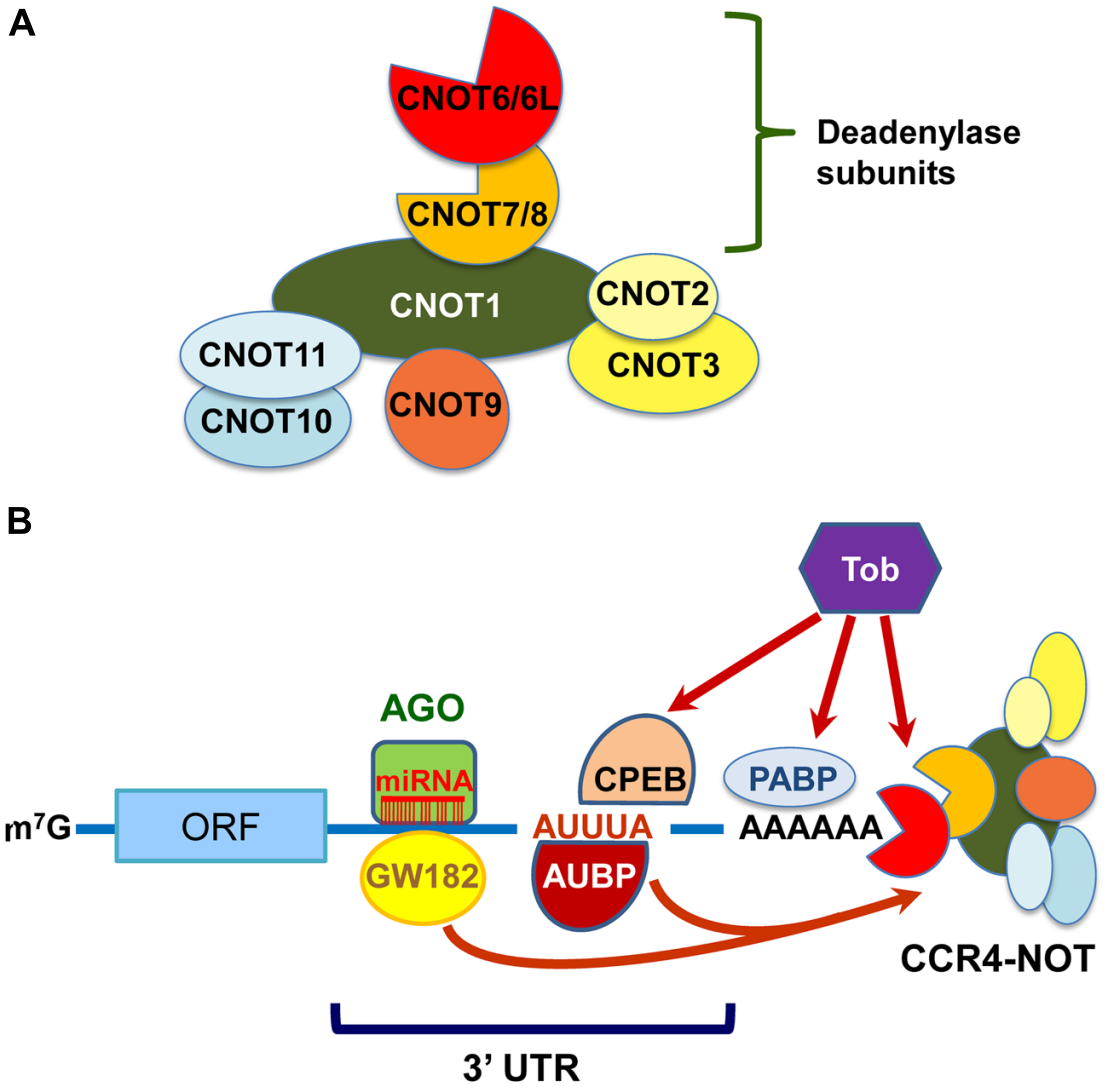
**The mammalian carbon catabolite repression 4 (CCR4)–negative on TATA-less (NOT) complex exerts deadenylase activities guided by RNA-binding proteins, miRISC and Tob. (A)** Schematic structural organization of the mammalian CCR4–NOT complex. It comprises either of CNOT6/6L and either of CNOT7/8 as deadenylase subunits. **(B)** Schematic representation of deadenylation by the mammalian CCR4–NOT complex. The CCR4–NOT complex is recruited to the 3′UTR of specific mRNAs through an interaction with AU-rich RNA binding proteins (AUBP) including TTP. GW182 is a component of miRISC together with miRNA and AGO. The CCR4–NOT complex is involved in the miRNA-induced deadenylation through an association with GW182. Tob also interacts with poly(A)-binding protein (PABP) and polyadenylation element-binding protein (CPEB), and recruits the CCR4–NOT complex to initiate deadenylation.

The CCR4–NOT components in yeast, except for Caf130p, are well conserved in mammals (Panepinto et al., 2013). CNOT1, CNOT2, CNOT4, and CNOT9 are mammalian orthologs of Not1p, Not2p, Not4p, and Caf40p, respectively. However, there are some compositional differences between the mammalian and yeast CCR4–NOT complexes. First, the E3 ubiquitin ligase CNOT4 does not stably associate with the mammalian CCR4–NOT complex (Lau et al., 2009). Second, there are four deadenylase subunits in the mammalian CCR4–NOT complex; yeast Ccr4p, which belongs to the exonuclease/endonuclease/phosphatase (EEP) family, is diverged into CNOT6 and CNOT6L, and Caf1p, which belongs to the DEDD (Asp-Glu-Asp-Asp) family, is into CNOT7 and CNOT8. The expression of these paralogs was reported to be mutually exclusive in the human CCR4–NOT complex in HeLa S3 cells (Lau et al., 2009). Third, CNOT3 is a mammalian ortholog of two yeast Not proteins: Not3p and Not5p. Finally, orthologs of mammalian CNOT10 and C2orf29 (CNOT11) are missing in fungi (Mauxion et al., 2013; Panepinto et al., 2013). As the function of some CCR4–NOT components is different between yeast and mammals, it would be interesting to compare their biological roles across species. The structural organization and deadenylation machinery of the mammalian CCR4–NOT complex can be proposed as shown in Figures 1A,B, respectively.

The CCR4–NOT complex is involved in various cellular mechanisms, including cell growth, DNA repair, mRNA export, histone methylation, and protein quality control in yeast (Collart, 2003; Mulder et al., 2005, 2007; Kerr et al., 2011; Halter et al., 2014). The CCR4–NOT complex is also implicated in the regulation of cell wall integrity, antifungal drug susceptibility, and developmental switches in pathogenic fungi (Panepinto et al., 2013). As we describe here, recent studies using cultured mammalian cells and genetically modified mice have revealed distinct roles of each subunit in wide-ranging physiological phenomena. Furthermore, deviation in the expression of CCR4–NOT has been found in various diseases based on analyses of human clinical specimens. In this review, we focus on the physiology of the mammalian CCR4–NOT complex and will occasionally refer to findings from non-mammalian systems.

## CNOT1

CNOT1 is the largest subunit of the CCR4–NOT complex in molecular size and is a scaffold protein involved in the assembly of CCR4–NOT components (**Figure 1A**). From a structural point of view, CNOT1 can be divided into three main regions, namely the *N*-terminal, the middle and the C-terminal, according to structural predictions and information (Bawankar et al., 2013). The *N*-terminal region of CNOT1 associates with CNOT11, which interacts with CNOT10 (Bawankar et al., 2013). The middle part of CNOT1, named the MIF4G (middle domain of eukaryotic initiation factor 4G) domain, directly interacts with CAF1a (CNOT7) or CAF1b (CNOT8; Basquin et al., 2012; Petit et al., 2012; Bawankar et al., 2013). In addition, CAF40 (CNOT9) interacts with the remaining residues of the middle portion of CNOT1, which comprises a DUF3819 domain (Bawankar et al., 2013; Chen et al., 2014; Mathys et al., 2014). Finally, the C-terminal region of CNOT1 associates with the CNOT2/CNOT3 complex (Bawankar et al., 2013; Boland et al., 2013). Through these interactions, CNOT1 provides a scaffold function for complex formation. Accordingly, depletion of CNOT1 destabilizes the complex, and each component is degraded, resulting in decreased levels of CNOT2, CNOT6L, CNOT7, and CNOT9, but not CNOT3, in HeLa cells (Ito et al., 2011a).

CNOT1 is an indispensable component for the deadenylase activities of the CCR4–NOT complex; however, CNOT1 does not possess any catalytic domains. Indeed, depletion of CNOT1 in HeLa cells compromises the deadenylase activities and decreases the number of P-bodies (Ito et al., 2011a), where mRNA decay is thought to take place (see review by Parker and Sheth, 2007). CNOT1-depleted cells undergo caspase-dependent apoptosis most likely through endoplasmic reticulum (ER) stress resulting from protein overproduction (Ito et al., 2011a).

CNOT1 also serves as a platform for the RNA-binding proteins that determine the specificity of the CCR4–NOT deadenylase for target mRNAs (**Figure 1B**). TTP, which is involved in AU-rich element (ARE)-mediated mRNA decay, directly interacts with CNOT1, and this interaction is required for the recruitment of CNOT7 to target mRNAs such as tumor necrosis factor-α (TNF-α) to promote deadenylation (Sandler et al., 2011; Fabian et al., 2013). In addition, mammalian Nanos paralogs (Nanos1, Nanos2, and Nanos3) directly interact with the C-terminal domain of CNOT1 (Suzuki et al., 2012; Bhandari et al., 2014). The interaction between Nanos2 and CNOT1 plays an important role in murine male germ cell development, most likely through regulation of the turnover of specific mRNAs, such as *Stra8* (Suzuki et al., 2010, 2012). CNOT1 was also reported to interact with IGF2 mRNA-binding protein 1 (IGF2BP1, also known as IMP1; Hämmerle et al., 2013). In human liver cancer cells, CCR4–NOT takes part in the posttranscriptional repression of the long noncoding RNA *h*ighly *u*p-regulated in *l*iver *c*ancer (*HULC*), to which IGF2BPs specifically bind (Hämmerle et al., 2013).

CNOT1 is a key player in linking the CCR4–NOT complex to miRNA-mediated repression (**Figure 1B**). Human GW182 proteins (also known as TNRC6A, TNRC6B, and TNRC6C), Argonaute (AGO)-binding partners and crucial effectors of miRISCs (Fabian and Sonenberg, 2012), were shown to directly interact with CNOT1 as well as PAN3 (Braun et al., 2011; Chekulaeva et al., 2011; Fabian et al., 2011). The GW182 proteins provide a platform for CCR4–NOT and PAN2–PAN3 to cause deadenylation of miRNA–target mRNAs (Braun et al., 2011; Chekulaeva et al., 2011; Fabian et al., 2011). In addition, CCR4–NOT is also required for silencing unadenylated RNA (Braun et al., 2011; Chekulaeva et al., 2011). Recent studies showed that the RNA helicase DDX6, a translational repressor and decapping activator, also interacts with CNOT1 through the MIF4G domain and this interaction is important for miRNA repression (Chen et al., 2014; Mathys et al., 2014). These findings propose that CCR4–NOT also plays an important role in miRNA-mediated translational suppression via recruitment of DDX6.

CNOT1 is commonly involved in mRNA decay in eukaryotes, including *Drosophila melanogaster* (Eulalio et al., 2007; Temme et al., 2010) and *Schmidtea mediterranea* (freshwater planarian) stem cells (Solana et al., 2013). Knockdown of planarian NOT1 increases stem cell specific-mRNAs with elongated poly(A) tails and leads to failure of cell differentiation (Solana et al., 2013). Remarkably, a single deletion of only the *NOT1* gene among the CCR4–NOT components causes lethality in *Saccharomyces cerevisiae*, while double deletion of the *CCR4* and *CAF1* genes does not cause lethality (Maillet et al., 2000), which also suggests that CNOT1 provides not only a scaffold function for the CCR4–NOT deadenylase complex but also multiple other functions that are important for cellular homeostasis.

Transcriptional regulation is considered one of the most important functions of CNOT1, as Not1p is known to regulate transcription by RNA polymerase II in yeast (Lee et al., 1998). In MCF-7 human breast cancer cells that are positive for estrogen receptor α (ERα), CNOT1 interacts with ERα through the LXXLL motif and represses the ligand-dependent transcriptional activation of ERα (Winkler et al., 2006). CNOT1 can also interact with another member of the nuclear receptor superfamily, retinoid X receptor α (RXRα), and suppress RXR-mediated transcription in a ligand-dependent fashion (Winkler et al., 2006). In addition, CNOT1 as well as CNOT2 and CNOT3 suppresses the expression of trophectoderm transcription factors and plays a critical role in maintaining the state of human and mouse embryonic stem cells (ESCs), contrary to what occurs in planarian stem cells (Zheng et al., 2012; See details in Section “CNOT2 and CNOT3”). A genome-wide RNAi screen identified CNOT1 and CNOT2 as transcriptional and/or higher-order regulators of major histocompatibility complex class II molecules (MHC-II) in human melanoma MelJuSo cells (Paul et al., 2011).

Some clinical studies indicate that *CNOT1* single nucleotide polymorphisms (SNPs) are relevant to physiological function, carcinogenesis, and the clinical outcome of disease. A locus in chromosome 16q21, which includes an intronic SNP within the *CNOT1* gene, was newly identified as a modulator of the QT interval duration of the electrocardiogram in two independent studies (Newton-Cheh et al., 2009; Pfeufer et al., 2009). *NOT1* was also identified as a key factor that was essential for heart functions using a genome-wide *in vivo* RNAi screen in *Drosophila* (Neely et al., 2010). In addition, SNPs within the *CNOT1* and *CNOT6* genes are associated with susceptibility to B-cell pediatric acute lymphoblastic leukemia (ALL; Gutierrez-Camino et al., 2014). The *CNOT1* SNP in dendritic cells (DCs) of 18 HIV patients was also associated with the response to DC-based immune therapy, as determined based on the plasma viral load (Moura et al., 2014). These findings suggest that CNOT1 is deeply involved in the functions of the cardiovascular, hematopoietic, and immune systems in humans.

Finally, *Cnot1* knockout (KO) mice died during embryonic development (unpublished). Analyses of *Cnot1* KO mice and heart or immunocyte-specific *Cnot1* conditional KO (cKO) mice would be an attractive strategy to clarify the molecular mechanism by which CNOT1 contributes to the maintenance of proper physiological functions in mammals. Findings from further studies are awaited.

## CNOT2 AND CNOT3

CNOT2 and CNOT3 are structurally similar, having a unique conserved domain, i.e., the NOT-box, which is localized at the C-termini of CNOT2 and CNOT3 (Zwartjes et al., 2004). The NOT-box of CNOT2 is involved in transcriptional regulation through an interaction with histone deacetylase 3 (HDAC3), which is associated with the retinoic acid (RA) receptor, thyroid-hormone receptor (SMRT), or nuclear hormone receptor co-repressor (NCoR; Zwartjes et al., 2004; Jayne et al., 2006). The CNOT2 and CNOT3 proteins directly interact with each other in various eukaryotes (Ito et al., 2011b; Bawankar et al., 2013; Boland et al., 2013), and this interaction is achieved through their C-terminal regions comprising the NOT-boxes (Bawankar et al., 2013; Boland et al., 2013). In addition, CNOT2 mediates the interaction between CNOT3 and CNOT1 (Lau et al., 2009; Ito et al., 2011b; Boland et al., 2013). Consistently, depletion of CNOT2 in HeLa cells affects the integrity of the CCR4–NOT complex. Its depletion extensively decreases the expression level of CNOT3 and modestly decreases the expression of other CNOTs by destabilizing them (Ito et al., 2011b).

CNOT2 and CNOT3 do not possess catalytic domains, and there has been no report about their direct enzymatic activities. However, increasing evidence indicates that CNOT2 and CNOT3 function as positive modulators of the catalytic activities of the CCR4–NOT deadenylase and promote the degradation of target mRNAs. For instance, depletion of Not2p, Not3p, or Not5p suppresses cellular deadenylase activity, albeit slightly, and results in the stabilization of mRNAs in yeast (Tucker et al., 2002). It is noteworthy that this function of CNOT2 and CNOT3 is pertinent to various biological phenomena. A *NOT2* or *NOT5* mutation is synthetically lethal with a *CAF1* or *CCR4* deletion (Bai et al., 1999). By contrast, a *NOT3* mutation with either a *CAF1* or *CCR4* deletion does not result in synthetic lethality (Bai et al., 1999), suggesting that Not5p is more essential than Not3p for the function of the CCR4–NOT complex in yeast.

Also, in higher eukaryotes, CNOT2 and CNOT3 regulate mRNA decay in various physiological processes. In *Drosophila* S2 cells, depletion of NOT2 affects the length of mRNA poly(A) tails (Temme et al., 2004). In addition, NOT3 directly interacts with the RNA-binding protein Bic-C, which is required for maternal patterning in the *Drosophila* embryo, and thus contributes to recruitment of the catalytic subunit of the deadenylase to its target mRNAs (Chicoine et al., 2007). In mammals, CNOT3 is essential for embryonic viability because deficiency in CNOT3 results in lethality of mice at early embryonic stages (Neely et al., 2010; Morita et al., 2011). Moreover, *Cnot3*^+/-^ mice are lean and display resistance to high-fat-diet (HFD)-induced obesity compared to control mice due to enhanced metabolic rates and glucose tolerance (Morita et al., 2011). We clarified that a reduction in CNOT3 expression affects the stability of specific mRNAs encoding proteins mainly involved in energy metabolism, such as pyruvate dehydrogenase kinase, isozyme 4 (PDK4), and IGF binding protein 1 (IGFBP1), in the livers of *Cnot3*^+/-^ mice (Morita et al., 2011). *Cnot3*^+/-^ mice also show osteopenia and exacerbated aging-induced osteoporosis with enhanced bone resorption (Watanabe et al., 2014). It was also found that the mRNA expression of receptor activator of nuclear factor-κB (*RANK*), but not RANK ligand (*RANKL*), is up-regulated in *Cnot3*^+/-^ mice and its mRNA stability is enhanced by *Cnot3*-deficiency in bone marrow-derived macrophages (Watanabe et al., 2014). These findings suggest that CNOT3-mediated regulation of the CCR4–NOT deadenylase is essential for a wide-range of physiological processes in mice.

In cultured mammalian cells, CNOT2 and CNOT3 reportedly affect cell cycle progression through the regulation of mRNA turnover. Knockdown of CNOT2 in HeLa cells impairs the deadenylase activity of the CCR4–NOT complex and decreases the number of P-bodies, as is the case for CNOT1 (Ito et al., 2011a,b). The cells, in turn, undergo caspase-dependent apoptosis, most likely due to the ER-stress induced by overproduction of the proteins that results from elevated levels of the stabilized mRNAs (Ito et al., 2011a,b). By contrast, knockdown of CNOT3 specifically increases the expression of MAD1 by stabilizing its mRNA and induces mitotic arrest of HeLa cells (Takahashi et al., 2012).

Transcriptional regulation is another important function of CNOT2 and CNOT3. Recently, a CNOT2-centered transcriptional network module was reported to be relevant for the susceptibility of breast cancer to metastasis (Faraji et al., 2014). CNOT2 also regulates the metastasis of murine breast cancer in a mouse xenograft model (Faraji et al., 2014). Regarding CNOT3, a genome-wide RNAi screen identified CNOT3 as one of the molecules that form a unique module in the transcriptional network and are essential for mouse ES cell self-renewal (Hu et al., 2009). CNOT2 and CNOT3 as well as CNOT1 contribute to the maintenance of mouse and human ESC identity, namely self-renewal and pluripotency, possibly through suppressing the expression of trophectoderm transcription factors (Zheng et al., 2012). Consistent with this, the expression of CNOT1, CNOT2, and CNOT3 closely correlates with the state of pluripotency (Zheng et al., 2012). It is noteworthy that single or even combined silencing of the deadenylase subunits does not affect the differentiation of ESCs, suggesting that transcriptional repression by CNOT1/CNOT2/CNOT3 is a key function in this phenomenon (Zheng et al., 2012). A global *in vivo Drosophila* RNAi screen identified NOT3 in addition to NOT1 as a critical regulator of heart function (Neely et al., 2010). In fact, *Cnot3*^+/-^ mice exhibit spontaneous impairment of cardiac contractility and develop severe cardiomyopathy in response to artificial cardiac stress by transverse aortic constriction (TAC) surgery (Neely et al., 2010). These phenomena are most likely due to decreased CNOT3 expression, resulting in the dysfunction of chromatin modification at the promoter regions of genes relevant to heart functions (Neely et al., 2010). However, in this case, it remains to be investigated whether CNOT3 also affects the stability of specific mRNAs in the heart (Neely et al., 2010).

Finally, several reports suggest that dysregulation of CNOT3 expression is relevant to a wide-range of human diseases. SNP analysis revealed that there is a significant association between CNOT3 polymorphism and cardiac repolarization duration (Neely et al., 2010) or susceptibility to ankylosing spondylitis (Díaz-Peña et al., 2012). In addition, CNOT3 was identified as a modifier of mutations in the causative gene of retinitis pigmentosa, *PRPF31*, which prevents the manifestation of this disease (Venturini et al., 2012). Importantly, CNOT3 was also identified as a tumor suppressor that is mutated in adult T-cell ALL, and its knockdown indeed caused tumors in a sensitized *D. melanogaster* model (De Keersmaecker et al., 2013). The mechanisms by which CNOT3 contributes to the prevention of carcinogenesis are unknown. As such, further studies are necessary to clarify the molecular functions of CNOT3, especially by addressing which of the two major mechanisms, mRNA decay or transcriptional regulation, mainly contributes to individual biological phenomena.

## CNOT6 AND CNOT6L

The evolutionarily conserved protein CCR4 is one of the two deadenylase subunits of the yeast CCR4–NOT complex and belongs to the EEP superfamily (Collart and Panasenko, 2012; Winkler and Balacco, 2013). Yeast CCR4 (Ccr4p) was originally identified as a regulator of ADH2 in the yeast *Saccharomyces cerevisiae* (Denis, 1984). In a series of genetic studies, Ccr4p was shown to be involved in biological functions such as the DNA damage response (Collart and Panasenko, 2012).

CCR4 is characterized by the presence of two highly conserved domains: a *C*-terminal deadenylase domain and an amino-terminal leucine-rich repeat (LRR) domain. The LRR domain is essential for binding to CAF1 and incorporation into the CCR4–NOT complex (Winkler and Balacco, 2013). However, there are some differences in the structure and composition of CCR4 among species. First, the activation domain located in the *N*-terminal-most region of *Saccharomyces cerevisiae* Ccr4p is not present in its orthologs in other species of eukaryotes, including mammals (Morita et al., 2007). Second, there is only a single CCR4 in yeasts as well as in the metazoans *D. melanogaster* and *Caenorhabditis elegans*, while two orthologs, CNOT6 (also known as CCR4a) and CNOT6L (also known as CCR4b), exist in vertebrates, including *Xenopus laevis*, *Danio rerio*, *Mus musculus*, and *Homo sapiens* (Dupressoir et al., 2001; Morita et al., 2007; Cooke et al., 2010; Winkler and Balacco, 2013).

CCR4 has been implicated in a variety of biological functions, as inferred from genetic and RNA interference experiments in many biological species (Goldstrohm and Wickens, 2008). It is worth noting that CCR4 family proteins are not essential for the viability of the organisms studied to date (Goldstrohm and Wickens, 2008). Among the various physiological roles of CCR4 over multiple species, the role of CCR4 during the early development of various organisms has been well characterized. For instance, mutations in the *Drosophila* gene *twin*, which encodes CCR4, affect female reproductive capacity and display defects in the self-renewal of germline stem cells (Temme et al., 2004; Morris et al., 2005; Zaessinger et al., 2006; Joly et al., 2013). In particular, the expression of the *mei-P26* mRNA is specifically regulated by CCR4 through the RNA-binding proteins Nanos and Pumilio during germline stem cell self-renewal in *Drosophila* (Joly et al., 2013). By contrast, the poly(A) tail length of mRNAs was globally increased by depletion of CCR4 in *C. elegans* germ cells, suggesting the CCR4–NOT complex plays a key role in general mRNA metabolism during early development in *C. elegans* (Nousch et al., 2013).

The mammalian CCR4 orthologs CNOT6 and CNOT6L have also been recently studied. The crystal structural analysis of human CNOT6L revealed that its deadenylase domain is remarkably similar to that of other EEP proteins and selectively interacts with poly(A) residues (Wang et al., 2010). In addition, *in vitro* deadenylation assays revealed that CNOT6L prefers poly(A) RNA to poly(A) DNA, indicating that CNOT6L preferentially functions as deadenylase of mRNAs (Wang et al., 2010).

CNOT6 and CNOT6L were reported to be mutually exclusive in the human CCR4–NOT complex in HeLa S3 cells, implying that the two paralogs have compensatory roles in mRNA deadenylation (Lau et al., 2009). Indeed, both CNOT6 and CNOT6L contribute to efficient cell growth of MCF-7 cells (Mittal et al., 2011). It should be noted that microarray analysis demonstrated that depletion of CNOT6 has little effect on mRNA levels compared to depletion of CNOT6L (Mittal et al., 2011). In addition, the tissue distribution of CNOT6 and CNOT6L in mice is distinctive. CNOT6L is ubiquitously expressed in various tissues, while CNOT6 is highly expressed in testis, ovary, thymus, and spleen (Chen et al., 2011). Moreover, CNOT6L, but not CNOT6, influences cell proliferation of murine fibroblasts (NIH3T3 cells) through regulation of the *Cdkn1b* mRNA (Morita et al., 2007). These findings indicate that CNOT6 and CNOT6L have discrete roles in addition to a mutually overlapping role in cells. Both *Cnot6* KO and *Cnot6l* KO mice are viable and fertile (unpublished). Further studies with these KO mice would unravel the physiological differences between CNOT6 and CNOT6L.

Recent study demonstrated that significant copy number loss of *CNOT6L* was present in human colon adenocarcinoma samples (Tay et al., 2011). The expression of CNOT6L and CNOT7 was reportedly down-regulated in samples of leukemia cells taken from ALL and AML (acute myeloid leukemia) patients compared to normal blood cells, while the expression of CNOT6 was slightly up-regulated (Maragozidis et al., 2012). In addition, SNPs in the *CNOT1* and *CNOT6* genes were significantly associated with B-cell ALL susceptibility (Gutierrez-Camino et al., 2014). However, the involvement of CNOT6 and CNOT6L in the onset and progression of cancer is still not clarified. Apparently, further studies investigating the function of CNOT6 and CNOT6L in various diseases are required.

Finally, there are additional members of the mammalian CCR4 family: Nocturnin (CCR4c), Angel1 (CCR4d), Angel2 (CCR4e), and 2′phosphodiesterase (2′PDE; Goldstrohm and Wickens, 2008). These proteins lack a LRR domain, which indicates that they are not always components of the CCR4–NOT complex (Godwin et al., 2013). Their physiological functions are still largely unknown, and we would like to omit these details in this review (see Poulsen et al., 2011; Godwin et al., 2013).

## CNOT7 AND CNOT8

Caf1, which possesses a ribonuclease (RNase) D domain from the DEDD superfamily, is the other core deadenylase subunit of the CCR4–NOT complex (Collart and Panasenko, 2012; Winkler and Balacco, 2013). Initially, yeast Caf1 (Caf1p) was identified as a possible transcriptional regulator required for glucose-derepression in *Saccharomyces cerevisiae* (Sakai et al., 1992). Subsequently, Tucker et al. found that Caf1p together with Ccr4p is a major cytoplasmic mRNA deadenylase (Tucker et al., 2001). Since then, deadenylation by Caf1 has been intensively studied.

The amino acid sequences of CAF1 orthologs are highly conserved across species (Winkler and Balacco, 2013). In *Trypanosoma brucei*, no ortholog for CCR4 was found, and CAF1 is the only deadenylase unit of the trypanosome CCR4–NOT complex, which is crucial for the deadenylation and subsequent degradation of many mRNAs (Schwede et al., 2008). In *Drosophila*, although both CCR4 and CAF1 are involved in the regulation of bulk mRNA poly(A) deadenylation, the catalytic activity of Caf1 is considered more potent than that of CCR4 (Temme et al., 2004, 2010). By contrast, Ccr4p is the dominant deadenylase, and Caf1p is dispensable in the presence of the catalytic activity of Ccr4p in yeast (Tucker et al., 2002). However, there remains a possibility that Caf1p is responsible for the regulation of deadenylation of specific mRNAs in yeast (Tucker et al., 2002; Wiederhold and Passmore, 2010). Thus, the biological significance of CAF1 differs among eukaryotes, even though the molecular structures of its orthologs are well conserved.

In mammals including humans, there are two orthologs of CAF1: CNOT7 (hCAF1a) and CNOT8 (hCAF1b). Amino acid sequence analysis of human CNOT7 and CNOT8 demonstrated that there is a high degree of homology between them (Bianchin et al., 2005; Winkler and Balacco, 2013). Human CNOT7 and CNOT8 bind the middle region of CNOT1 through its MIF4G domain, which is composed of antiparallel pairs of α-helices (Basquin et al., 2012; Petit et al., 2012; Bawankar et al., 2013). In addition, yeast two-hybrid analysis revealed that CNOT6 directly binds CNOT7 and CNOT8, but not CNOT1 (Lau et al., 2009). These findings suggest that the interaction of CNOT7 and CNOT8 with CNOT1 is essential for CNOT6 and CNOT6L to be integrated into the CCR4–NOT complex and exert their deadenylation functions.

Both CNOT7 and CNOT8 are ubiquitously expressed in adult mouse tissues (Chen et al., 2011). It is worth mentioning that high expression levels of both CNOT7 and CNOT8 proteins were detected, especially in thymus, spleen, ovary, and testis, and their expression patterns were similar (Chen et al., 2011). The combined depletion of CNOT7 and CNOT8 as well as CNOT6 and CNOT6L resulted in the strong inhibition of ARE-containing β-globin mRNA decay in human HTGM5 cells (Schwede et al., 2008). In addition, CNOT7 and CNOT8 were reported to be mutually exclusive in the CCR4–NOT complex in HeLa S3 cells, as is the case for CNOT6 and CNOT6L (Lau et al., 2009). These findings suggest that the Caf1 paralogs are important for mRNA deadenylation in mammals, and each has overlapping functions. Indeed, CNOT7 and CNOT8 are required for the proliferation of MCF7 human breast cancer cells and redundantly repress the antiproliferative gene *PMP22* through their deadenylase activities (Aslam et al., 2009).

It is noteworthy that CNOT7 and CNOT8 are capable of binding BTG/Tob proteins (BTG1, BTG2/PC3/Tis21, BTG3/ANA, BTG4/PC3B, Tob1, and Tob2), which are characterized by the presence of a conserved BTG domain and regulate cell cycle progression (Winkler, 2010; Winkler and Balacco, 2013). The interaction with CNOT7/CNOT8 is required for the anti-proliferative activities of Tob proteins in various types of mammalian cells (Horiuchi et al., 2009; Doidge et al., 2012b; Ezzeddine et al., 2012). Importantly, Tob functions as a guide molecule for the CCR4–NOT complex to target mRNAs through an interaction with CNOT7 (Doidge et al., 2012a). Tob is capable of associating with the general RNA-binding protein, poly(A)-binding protein (PABP), and recruits the CCR4–NOT complex to initiate mRNA decay (Funakoshi et al., 2007). Moreover, Tob also mediates the recruitment of the CCR4–NOT deadenylase to specific mRNAs, such as *MYC*, to which CPEB proteins bind (Hosoda et al., 2011; Ogami et al., 2014). There are some reports that Tob proteins and BTG2 activate the deadenylation of mRNAs in cultured cells (Ezzeddine et al., 2007, 2012; Mauxion et al., 2008). However, there is an opposing report that BTG2 inhibits deadenylation by CNOT7/CNOT8 *in vitro* (Yang et al., 2008). As such, regulation of the deadenylation activity of CNOT7/CNOT8 by BTG/Tob proteins might be dependent on extracellular and intracellular circumstances. Further studies should clarify this complicated molecular network.

CNOT7 is known to be deeply involved in cellular signaling pathways through interacting with various types of proteins in addition to BTG/Tob proteins. CNOT7 regulates class I and II interferon (IFN) pathways by controlling signal transducer and activator of transcription 1 (STAT1) trafficking through an interaction with its latent form and degrading STAT1-regulated mRNAs through its deadenylase activity (Chapat et al., 2013). Remarkably, *Cnot7*-deficiency results in physiological anomalies in mice. *Cnot7* KO male mice exhibit severe infertility associated with oligo-astheno-teratozoospermia (Berthet et al., 2004; Nakamura et al., 2004). CNOT7 physically interacts with RXR beta (Rxrb) and is involved in spermatogenesis by functioning as a coregulator of Rxrb in testicular somatic cells (Nakamura et al., 2004). Moreover, *Cnot7* KO mice show increased bone mass, and CNOT7 was found to function as a suppressor of bone morphogenetic protein (BMP) signaling (Washio-Oikawa et al., 2007). These findings suggest that CNOT7 regulates the balance in various biological processes in mammals. Both *Cnot7* and *Cnot8* KO mice would be useful tools to clarify the functional difference between CNOT7 and CNOT8.

There are some reports that CNOT7 and CNOT8 are relevant to the development and progression of cancer. The expression of CNOT7 was down-regulated in ALL and AML patients (Maragozidis et al., 2012), and the expression of CNOT8 was elevated in primary colorectal carcinoma and metastatic legions compared to the normal mucosa (Seiden-Long et al., 2006). It remains to be investigated how CNOT7 and CNOT8 contribute to the development and progression of cancer.

## CNOT9

Rcd1 (required for cell differentiation 1) was originally identified as a factor essential for nitrogen starvation-induced sexual differentiation in the fission yeast *Schizosaccharomyces pombe* (Okazaki et al., 1998). Shortly after this discovery, its *Schizosaccharomyces cerevisiae* homolog, Caf40, was identified by mass spectrometry analysis as a component of the CCR4–NOT complex (Chen et al., 2001). The amino acid sequences of Rcd1/Caf40 orthologs are highly conserved among eukaryotes (Okazaki et al., 1998; Garces et al., 2007).

CNOT9, the mammalian ortholog of Rcd1 and Caf40 (Collart and Timmers, 2004), acts as a transcriptional cofactor that plays a critical role in RA-induced differentiation of F9 embryonic teratocarcinoma cells and in lung development, through an association with a transcription complex containing activation transcription factor-2 (ATF-2) and RA receptors (RARs) in an RA-dependent fashion (Hiroi et al., 2002). CNOT9 interacts with nuclear hormone receptor transcriptional coactivator (NRC)-interacting factor 1 (NIF-1; Garapaty et al., 2008). NIF-1 interacts with NRC, which is a coactivator of nuclear hormone receptors, and regulates its activities (Mahajan et al., 2002). CNOT9 also interacts with c-Myb, which is a proto-oncogene product and transcription factor involved in myeloid cell differentiation (Haas et al., 2004). In addition, CNOT9 was isolated as an erythropoietin-responsive gene that is potentially important for hematopoietic cell development (Gregory et al., 2000). Thus, the involvement of CNOT9 in the control of growth and differentiation of mammalian tissues, especially of the hematopoietic system, may be highlighted.

Although CNOT9 has not been shown to carry deadenylase activity, its involvement in deadenylation and/or mRNA decay was recently implicated by structural analysis (Chen et al., 2014; Mathys et al., 2014). CNOT9 comprises six armadillo repeats (ARM repeat domain) and forms a homodimer or alternatively interacts with CNOT1 (Garces et al., 2007; Chen et al., 2014; Mathys et al., 2014). The interaction takes place between the ARM repeat domain of CNOT9 and the DUF3819 domain of CNOT1, and the consequent binary complex provides binding sites for GW182 (Chen et al., 2014; Mathys et al., 2014). The ARM repeat domain is known to be involved in protein–protein interactions, as described above. Interestingly, a recent study revealed that the ARM repeat domain of β-catenin is associated with RNA binding and forming RNA–protein complexes (Kim et al., 2012), which suggests that CNOT9 is also capable of interacting with RNA through the ARM repeat domain. Consistently, a previous *in vitro* binding assay demonstrated the interaction between CNOT9 and single-stranded DNA polymers, i.e., poly(dC), poly(dG), and poly(dT), but not poly(dA) (Garces et al., 2007).

Acting as a transcriptional regulator and/or an mRNA silencing protein, CNOT9 is expected to play important roles in various biological events in mammals. Increasing data indicate that the aberrant expression of CNOT9 is relevant to disease, as described below. First, some evidence suggests that CNOT9 is involved in mammary carcinogenesis. CNOT9 is frequently up-regulated in human breast cancer specimens and cell lines (Ajiro et al., 2009), and its expression is extremely low in normal human tissues, except testis, which suggests that CNOT9 is a cancer antigen (Ajiro et al., 2009). It should be noted, however, that the expression of CNOT9 is readily detectable in various murine adult tissues, including testis, ovary, thymus, brain, and lung (Chen et al., 2011). Short hairpin RNA (shRNA)-mediated suppression of CNOT9 drastically suppresses the proliferation of breast cancer cells (Ajiro et al., 2009). In addition, CNOT9 forms a large complex consisting of Grb10, Grb10-interacting protein 1 (GIGYF1), GIGYF2, and Akt, all of which are components of epidermal growth factor receptor (EGFR) downstream signaling (Ajiro et al., 2010). The Akt phosphorylation that is induced by EGFR signaling is attenuated in the absence of CNOT9 (Ajiro et al., 2010), which suggests that CNOT9 enhances EGFR signaling and contributes to carcinogenesis and progression of cancer, particularly in mammary glands. Second, *Cnot9*-deficient mice are embryonic lethal (unpublished). Further analyses of *Cnot9* null and cKO mice would provide insights into how CNOT9 regulates physiological processes through interactions with various types of molecules.

## CNOT10 AND CNOT11

CNOT10 is a unique component of the CCR4–NOT complex in that its orthologs exist in eukaryotes, except in fungi (Bartlam and Yamamoto, 2010; Panepinto et al., 2013). Recent proteomic analysis uncovered that C2orf29 stably associates with the human CCR4–NOT complex (Lau et al., 2009). Ortholog of C2orf29 is also found in all eukaryotes examined except in yeast (Mauxion et al., 2013). In the vertebrate CCR4–NOT complex, C2orf29 together with CNOT10 form a module that interacts with CNOT1. This interaction takes place through direct binding of C2orf29 with the *N*-terminus of CNOT1 in *D. melanogaster* and human cells (Bawankar et al., 2013; Mauxion et al., 2013). In addition, expression of CNOT10 and C2orf29 reciprocally stabilizes each other. C2orf29 was proposed to be renamed CNOT11 and is now recognized as a bona fide subunit of the CCR4–NOT complex.

Regarding the function of CNOT10/CNOT11, its ability to promote deadenylation of bound mRNAs was shown in tethering assays to be most likely due to recruitment of the catalytic module (Bawankar et al., 2013). However, depletion of CNOT11 did not affect the deadenylation rate of a β-globin mRNA reporter in human cells (Mauxion et al., 2013), suggesting that CNOT11 itself is not directly involved in deadenylation. In contrast, depletion of CNOT10 impaired the deadenylation of various mRNAs and inhibited the proliferation of trypanosomes (Färber et al., 2013). This discrepancy in the role of CNOT10/CNOT11 might result from constitutional and structural differences between the human and trypanosome CCR4–NOT complexes. Indeed, depletion of CNOT10 decreased the level of CNOT1 in trypanosomes, while this phenomenon was not observed in human cells (Färber et al., 2013). This finding suggests that the role of the CNOT10/CNOT11 module could differ among species, even though the module is conserved. Finally, *Cnot10*-deficiency in mice causes embryonic lethality (unpublished). The generation and analyses of *Cnot11* KO mice would be necessary in future studies.

## CONCLUDING REMARKS AND PERSPECTIVES

The core members of the CCR4–NOT complex were originally identified and characterized in yeast (Collart, 2003). Recent studies, including structural and biochemical analyses, have unveiled that the mammalian CCR4–NOT complex harbors both conserved and mammalian-specific functions. In addition, each subunit has distinct multifunctional roles through interactions with a wide-range of molecules. By contrast, the deadenylase units, namely CNOT6/CNOT6L and CNOT7/CNOT8, have overlapping functions. The biological significance of the versatile and yet conserved roles, to a certain extent, has not been well appreciated, although this complexity makes research on the CCR4–NOT complex intriguing. Many questions remain to be addressed. For instance, what are the roles of CNOT10 and CNOT11 in the murine and human CCR4–NOT complexes? Strikingly, deficiency in each component of the CCR4–NOT complex, including CNOT1, CNOT3, CNOT9, and CNOT10, but not the deadenylase subunits CNOT6, CNOT6L, and CNOT7, in mice leads to embryonic lethality, suggesting that the CCR4–NOT complex is indispensable for embryonic development (unpublished). We are currently investigating whether *Cnot2* KO and *Cnot8* KO mice also exhibit developmental anomalies. Furthermore, the organ-specific functions of each CCR4–NOT component in mammals remain to be elucidated and could be addressed by generating cKO mice of each subunit in various organs. All the phenotypes of KO mice of each CCR4–NOT subunit reported are summarized in **Table 1**. Further analyses of null and cKO mice will help to clarify the inscrutable functions of the CCR4–NOT complex.

**Table 1:** Phenotypes of KO mice of the CCR4–NOT complex subunits.

Gene	Genotype	Dysregulation in the molecular functions	Biological anomaly	Reference
*Cnot3*	-/-	Unknown	Embryonic lethality (E6.5)	Neely et al. (2010), Morita et al. (2011)
	+/-	Up-regulation of *Pdk4* and *Igfbp1* mRNA	Leanness	Morita et al. (2011)
		Up-regulation of *Rank* mRNA	Osteopenia	Watanabe et al. (2014)
		Transcriptionally inactive chromatin	Cardiac dysfunction	Neely et al. (2010)
*Cnot7*	-/-	Impaired Rxrb signaling	Male sterility	Berthet et al. (2004), Nakamura et al. (2004)
		Enhanced BMP signaling	Increased bone mass	Washio-Oikawa et al. (2007)

In summary, the CCR4–NOT complex globally regulates mRNA metabolism from transcriptional initiation to mRNA degradation. Thus, the CCR4–NOT complex has great influence on biological events via the regulation of consequent protein expression. It is becoming clear that there is a relationship between the CCR4–NOT complex and various diseases. Dissection of the physiological processes that are regulated by CCR4–NOT, a mRNA metabolism-controlling machinery, would be a novel and intriguing approach to elucidate the cause of disease, both at onset and upon progression. Further progress in the research on mRNA metabolism regulated by CCR4–NOT is anticipated.

## Conflict of Interest Statement

The authors declare that the research was conducted in the absence of any commercial or financial relationships that could be construed as a potential conflict of interest.
